# AAV-mediated transduction of songbird retina

**DOI:** 10.3389/fphys.2025.1549585

**Published:** 2025-03-19

**Authors:** Pranav Kumar Seth, Dominik Heyers, Baladev Satish, Ezequiel Mendoza, Katrin Haase, Lisa Borowsky, Isabelle Musielak, Karl-Wilhelm Koch, Regina Feederle, Constance Scharff, Karin Dedek, Henrik Mouritsen

**Affiliations:** ^1^ Neurosensorics Group/Animal Navigation, Institute of Biology and Environmental Sciences, Carl on Ossietzky University of Oldenburg, Oldenburg, Germany; ^2^ Sussex Neuroscience, School of Life Sciences, University of Sussex, Brighton, United Kingdom; ^3^ Research Centre for Neurosensory Science, Carl von Ossietzky University of Oldenburg, Oldenburg, Germany; ^4^ Institut für Biologie, Freie Universität Berlin, Berlin, Germany; ^5^ Department of Neuroscience, Biochemistry Group, University of Oldenburg, Oldenburg, Germany; ^6^ Monoclonal Antibody Core Facility, Helmholtz Zentrum München, German Research Center for Environmental Health, Neuherberg, Germany

**Keywords:** AAV, avian retina, photoreceptors, intravitreal injection, European robin, opsin

## Abstract

**Introduction:**

Genetic manipulation of murine retinal tissue through ocular administration of adeno-associated viruses (AAVs) has become a standard technique to investigate a multitude of mechanisms underlying retinal physiology. Resultantly, developments of recombinant viral vectors with improved transduction efficiency and further methodological improvements have mostly focused on murine tissue, whereas AAVs successfully targeting avian retinae have remained scarce.

**Methodology:**

Using a custom-designed injection setup, we identified a viral serotype with the capability to successfully induce widespread transduction of the bird retina.

**Results:**

Intravitreal administration of an AAV type 2/9 encoding for enhanced green fluorescent protein (EGFP) in night-migratory European robins (*Erithacus rubecula*) resulted in transduction coverages of up to 60% within retinal tissue. Subsequent immunohistochemical analyses revealed that the AAV2/9-EGFP serotype almost exclusively targeted photoreceptors: rods, various single cones (UV, blue, green, and red cones), and both (accessory and principal) members of double cones.

**Discussion:**

The consistently high and photoreceptor-specific transduction efficiency makes the AAV2/9 serotype a powerful tool for carrying out genetic manipulations in avian retinal photoreceptors, thus opening a wealth of opportunities to investigate physiological aspects underlying retinal processing in birds, such as physiological recordings and/or post-transductional behavioural readouts for future vision-related research.

## Introduction

Adeno-associated virus (AAV) vectors are small, single-stranded DNA viruses displaying high transduction efficiency and tropism towards a wide range of host cells (Bennett et al., 1994; Jomary et al., 1994; Li et al., 1994). Their limited capability to induce immune responses has turned them into viable gene therapy tools (Atchison et al., 1965; Balakrishnan and Jayandharan; Ronzitti et al., 2020) for the investigation and treatment of retinal disorders (Buch et al., 2008; Stieger et al., 2011; Ong et al., 2019), such as, e.g., retinal degeneration and/or photoreceptor dystrophies (Ali et al., 2000; Schlichtenbrede et al., 2003; Isiegas et al., 2016; Ziccardi et al., 2019; Bacci et al., 2022).

Over the last 2 decades, mice have turned into the standard model system for retinal viral transduction experiments. Consequently, the continuously ongoing development of recombinant viral vectors with improved transduction efficiency has mostly targeted the murine visual system. In contrast, avian tissue appears to be particularly resistant to transduction using commonly available viral tools (Ahmadiantehrani and London, 2017). Resultantly, only few recombinant viral vector types have proven capable of transducing retinal tissue in birds (Scott and Lois, 2005; Harpavat and Cepko, 2006; Williams et al., 2006; Verrier et al., 2011; Vergara and Canto-Soler, 2012; Waldner et al., 2019).

Moreover, anatomical characteristics specific to birds appear to be disadvantageous to common ocular injection routes, further impeding successful transductions: the subretinal injection method, which targets the space between the photoreceptors and the retinal pigment epithelium (RPE) (Mühlfriedel et al., 2013; Park et al., 2015; Yiu et al., 2020), causes temporary focal detachment of the retina at the injection site (Cebulla et al., 2012; Waldner et al., 2019), leading to the formation of a “suspension bubble”. The subretinal space between the photoreceptors and the retinal pigment epithelium in birds is considerably smaller than in mice, thus, in addition to causing potential harm to retinal tissue within the bubble, it restricts the transduction in bird retinae to the near vicinity of the “suspension bubble” (Waldner et al., 2019).

Similarly, the suprachoroidal injection route, where the space between the sclera and the choroid is targeted for viral injections (Kansara et al., 2020; Yiu et al., 2020), is methodologically challenging in birds, since the vast majority of the avian eye remains hidden in the eye socket with only parts of the sclera being visible. This leaves the intravitreal injection route as the most feasible injection method in birds, where the viral suspension is directly injected into the vitreous chamber of the eye (Giove et al., 2010). This, however, has mainly resulted in low transduction efficiency in avian retinae with the currently available genetic tools (Waldner et al., 2019).

It is surprising that in the continuous development of new genetic tools and methodological improvements for retinal research in birds has been largely neglected over the last years. In particular because birds appear to be exceptionally well suited as model systems for vision-based research: (1) avian eyes occupy a major proportion of the head (Burton, 2008); (2) related visual brain parts occupy up to 50% of the total cranial capacity in certain bird species (Waldvogel, 1990; reviewed in Seifert et al., 2020); (3) the number of retinofugal fibers in birds outclasses that of man by a factor of 2.5 (Güntürkün et al., 1993); (4) several physiological aspects of avian vision, e.g., acuity, luminance detection and/or color discrimination easily surpass that of most mammals (Jones et al., 2007; Niu et al., 2022); (5) in contrast to the rod-dominated mouse retina (Jeon et al., 1998), many avian retinae contain foveae, i.e., areas of high cone photoreceptor density, also found in humans and other primates (Haverkamp et al., 2021). Here, we used the long-distance night-migratory European robin as a study species because its retina contains a light-dependent magnetic compass (Chetverikova et al., 2022; Günther et al., 2018; Wiltschko et al., 1993; Xu et al., 2021; Zapka et al., 2009). Moreover, its retina has recently been morphologically characterized using electron microscopy (Günther et al., 2024; 2025) and immunohistochemistry (Günther et al., 2018; Chetverikova et al., 2022; Balaji et al., 2023). These findings provide a good foundation for electrophysiological studies, which are currently rare (but see Rotov et al., 2022). Finding AAV serotypes that work in the European robin will therefore be a step forward in both the study of magnetoreception and the functional analysis of avian retinal circuits.

## Materials and methods

### AAV production

The generation of the plasmid was performed as described in Balaji et al. (2023). We used an AAV 2/9 serotype carrying a strong ubiquitous CAG/CAAG promoter and the enhanced green fluorescent protein (EGFP) as the fluorescent reporter. Its titer (CAG: 3.97 × 10^12^ VG/mL; (Balaji et al., 2023); CAAG: 1.77 × 10^12^ VG/mL) was quantified via genomic qPCR by the Viral Core Facility of Charité–Universitätsmedizin Berlin, Germany.

### Custom-designed ocular injection apparatus

**FIGURE 1:**
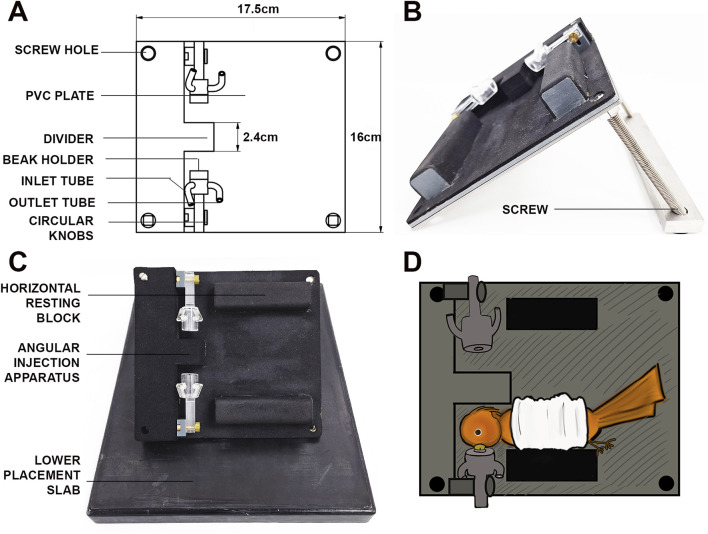
Custom-made ocular injection apparatus for intravitreal injections in birds. **(A)** Scheme of the angular injection apparatus, with the two beak holders attached to the PVC plate, and the inlet/outlet tubes attached to the beak holder. **(B)** Side view of the angular injection plate, depicting the beak holders, the inlet/outlet tubes and the metal screws for angular adjustment. **(C)** Front view of the angular injection plate, placed on top of the lower placement slab. **(D)** Schematic depiction of the placement of the experimental bird in the injection apparatus. Note the second beak holder in D can be used to inject the contralateral eye of the bird if need arises.

For carrying out the intravitreal injections, we used a custom-designed ocular injection apparatus, consisting of a placement slab, an angular injection unit and attached gas anesthesia delivery extensions (Figure 1A). The apparatus has been designed and constructed to meet the requirements of intravitreal injections in small passerines. The device was made with Poly Vinyl Chloride (PVC) and covered with Perbunan® which prevents unwanted loss of heat from the bird. The placement slab at the bottom was designed to provide stability to the above angular injection unit as well as resistance against sudden movements during the ocular injections (Figure 1C). The angular injection unit contained two beak holders placed on opposite sides to immobilize the bird on either side and to access both eyes easily during the injections.

The isoflurane gas used as an anaesthetic was provided to the birds with the help of a tube connecting the anesthesia device to an inlet in the beak holder. The outlet of the beak holder was connected with a suction pipe to remove excess anesthetic gas. Circular knobs attached to the beak holders enabled a movement along the antero-posterior axis to assist fixation of the bird’s head. A PVC block on either side was placed horizontally next to the beak holders to position the bird’s body on its side in a natural resting position while being anesthetized. The four corners of the ground plate had screw holes to enable the adjustment of the angle of the injection unit to the ground, using two long screws at a time. In our case, an angle of approximately 30° between the angular injection unit and the lower placement slab proved optimal for intravitreal injections (Figure 1B).

### Intravitreal injection protocol

Before each surgery, a single adult European robin was food-deprived for 2 h and fully anesthetized using Isoflurane CP® gas anesthesia (1 mL/mL; cp-pharma, Burgdorf, Germany) dissolved in oxygen; (2%–3% volume at initial stages of anesthesia, ∼1.5% volume throughout the surgery) directed through the beak holder. Meloxicam (Metacam®, Boehringer Ingelheim, Ingelheim, Germany; 0.2 mL/kg body weight dissolved in 0.9% NaCl) was administered intramuscularly for post-surgical analgesia. The bird was wrapped with a bandage cloth to prevent wing movement and placed in the custom-designed injection apparatus (Figure 1D). The anaesthetized bird´s head was carefully fixed by inserting the beak into the beak holder, and its eye lid was temporarily pulled back to get an unobstructed view of the eye. Additional local anaesthesia to the cornea was provided using Oxibuprocaine-hydrochloride. Upon locating the sclera-cornea junction at the dorso-temporal side of the eye with a stereoscope (Leica M400E, Wetzlar, Germany), a 27G needle was used to puncture the sclera, avoiding nearby blood capillaries from getting ruptured. This puncture was subsequently used as an entry point for the Hamilton syringe attached to a blunt 33G needle (VWR International GmbH, Germany), carrying the AAV2/9 viral suspension.

**FIGURE 2:**
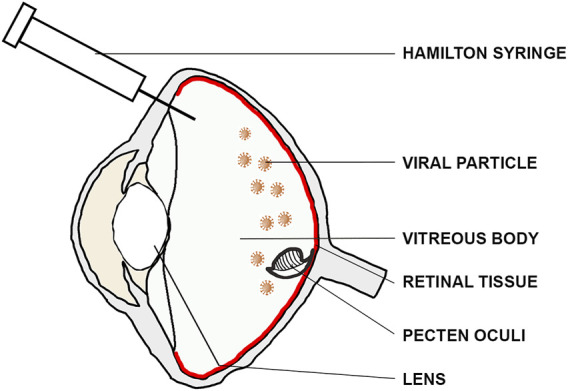
Intravitreal injection route. For intravitreal injections, the lens was bypassed using an insertion angle of ∼60–70° relative to the eye’s coronal axis to inject the viral suspension (brown) close to the retinal surface (red).

To minimize any unintentional damage to the bird’s vision, we carefully avoided the lens by using an insertion angle of ∼60°–70° relative to the eye’s coronal axis (Figure 2). Afterwards, the Hamilton syringe was inserted down to the fundus, and 10–20 µL of viral suspension was injected close to the retinal surface with an approximate speed of ∼1 μL/s. The syringe was held in place for at least 10 s after the injection in order to prevent reflux and to ensure dispersion of the viral suspension and carefully retracted afterwards. Post-surgery, the bird was taken out of the injection apparatus and transferred onto a warming plate for quick recovery. Each bird was monitored until it recovered from anesthesia and returned to its home cage upon gaining full consciousness. We provided post-surgical analgesia (Meloxicam administered intramuscularly; 0.2 mL/kg body weight dissolved in 0.9% NaCl) for up to 72 h post-surgery to minimize any signs of discomfort resulting from the surgery. For legal and ethical reasons, we did not perform vehicle-only controls as we had a strictly limited number of wild caught birds available. However, in order to validate the general functionality of the virus, we only injected one eye with the virus, while leaving the contralateral one as a negative control for subsequent immunostainings.

### Tissue processing

In line with previous studies on virus-mediated transduction of retinal tissue (Waldner et al., 2019; Nieuwenhuis et al., 2023), we let the AAV2/9 suspension reside inside the injected eye for 21–25 days, following which the birds were sacrificed by decapitation and the injected eye was dissected from the skull. The anterior part of the eye was removed along its coronal axis using a sharp razor blade and the vitreous body was carefully taken out. The eye cup was fixed using 4% paraformaldehyde [PFA dissolved in 0.1 M phosphate buffered saline, pH 7.6 (PBS)] for 30 min. Subsequently, the eye cups were washed three times in PBS for 15 min each and cryoprotected in a graded series of sucrose solutions (10%, 20%, 30% dissolved in 0.1 M PBS) overnight. If necessary, the eyecups were stored in 30% sucrose solution at −20°C until they were subjected to immunohistochemistry.

### Immunohistochemistry

Retinal tissue was cryosectioned on a freezing microtome (Leica CM 1860; Wetzlar, Germany) into serial cross sections with a thickness of 30 μm and placed onto microscope slides (epredia, Superfrost Plus Adhesion slides, Fisher Scientific, Waltham, MA, United States). For EGFP immunohistochemistry, the slides were briefly dried on a warming plate and washed twice with 0.1 M PBS for 15 min. Unspecific binding sites were blocked using 5% donkey serum (Sigma-Aldrich, Burlington, MA, United States) and 0.3%–0.5% Triton X-100 (Carl Roth, Germany) dissolved in 0.1 M PBS for 1–2 h. The slides were subsequently incubated overnight with a goat anti-GFP antibody (diluted 1:500 in blocking solution; 600-101-215; RRID: AB_218182; Rockland, Pottstown, PA, United States) together with one of the opsin antibodies listed in Table 1 at 4°C to assess the type of transduced photoreceptors. On the following day, slides were washed thrice for 10–15 min each in 0.1 M PBS. The retinal slices were subsequently incubated with appropriate secondary antibodies (Alexa Fluor 488-conjugated (anti goat) for EGFP; Alexa 568-conjugated (anti mouse) for rhodopsin; Alexa647-conjugated (anti guinea pig) for red opsin; Alexa 568-conjugated (anti mouse) for green opsin; Alexa 568 (anti rat) for blue opsin; Alexa 568 (anti rabbit) for UV opsin; dilution 1:500, Thermo Fisher Scientific, Waltham, MA, United States) for 2 h. The slices were washed thrice with PBS for 10–15 min and mounted with Vectashield mounting medium (containing nuclear DAPI stain; BIOZOL, Germany).

**TABLE 1:** Opsin antibodies used in this study.

Opsin antibody	Clone/company/catalogue number	Host species	Dilution	Immunogen sequence
Rhodopsin	Clone 1D4/Cell essentials/ab5417	Mouse	1:500	Detailed sequence not provided by the manufacturer
Red opsin	Karl W. Koch lab/Davids Biotechnologies	Guinea pig	1:1,000	SRYWPHGLKTSCGPDVFSGSSDPGVQSYMVSI
Green opsin	OPSG2 clone 26G5/Helmholtz Munich	Mouse	1:500	GPDYYTHNPDFH
Blue opsin^a^	OPSB clone 2D6/Helmholtz Munich	Rat	1:5	MHPPRPTTDLPEDF
UV opsin (Opsin, blue)	Millipore/AB5407	Rabbit	1:500	Recombinant human blue opsin

^a^ BLUE opsin (OPSB) (Günther et al., 2018).

### Image acquisition, processing, and quantification

**FIGURE 3:**
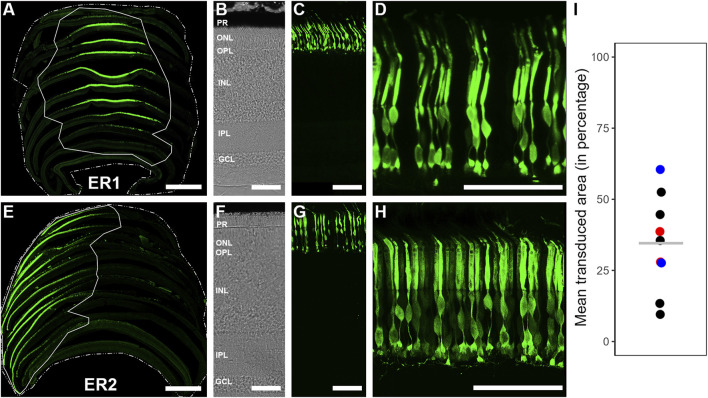
EGFP transduction within European robin retinae following intravitreal injection of AAV2/9-CAG-EGFP/AAV2/9-CAAG-EGFP. **(A, E)** 2-D reconstruction and determination of the transduction extent on two exemplary retinae, using single retinal sections placed in their respective order, displaying mean retinal transduction coverages of ∼28% (ER1) and ∼39% (ER2), respectively. Scale: 1 mm. **(B, F)** Transmission images of European robin retinae ER1 and ER2, respectively, depicting the retinal stratification used for anatomical orientation. Scale: 50 μm. **(C, G)** Vertical sections of European robin retinae ER1 and ER2 depicting the transduction success exclusive to the photoreceptor layer in most cases. Scale: 50 μm. **(D, H)** Zoomed-in images of the photoreceptor layer from European robin retinae ER1 and ER2, proving successful transduction of photoreceptors. Scale: 50 μm. **(I)** Mean transduced area (in %) of all transduced retinae. ER1 (∼28%) and ER2 (∼39%) are depicted by red circles, black circles depict the other retinal transductions using AAV2/9-CAG-EGFP; blue circles depict two cases where AAV2/9-CAAG-EGFP was used. Note the very similar transduction efficiency irrespective of the promotor type. The overall mean transduction coverage of 35% ± 17% (n = 9) is represented by a grey horizontal line.

Retinal sections were imaged with a confocal laser scanning microscope (Leica TCS SP8, Leica Microsystems, Wetzlar, Germany), using a HC PL APO 40x/1.3 or HC PL APO 63x/1.4 oil immersion objective. We used the “Navigator” software (LAS X Life Science, Wetzlar, Germany) to image retinal sections from each transduced eye. Twelve to fourteen retinal sections across the entire eye cup were used to determine the transduction efficiency in each transduced eye. Each section was individually analyzed as follows: 1) We normalized the intensity using the *Contrast Enhancement* tool (0.2%) in Fiji (Schindelin et al., 2012). 2) Both the transduced area and the total retina area on each section were individually marked using the *Freehand* tool. 3) The *Measure* tool was used to obtain the respective area sizes, and the fraction of transduced area was calculated for each individual section. To estimate the transduction efficiency per total retina, we used spline extrapolation to predict the values of the slides interjacent to the analyzed ones. We then averaged all values to calculate the fraction of transduced retina area. Figures 3C, D, G, H display the series of multiple Z-stack images (0.27 μm step size) merged into a maximum projection image. High-resolution scans of EGFP-labeled photoreceptors were normalized in Fiji using the *Contrast Enhancement* function.

## Results

### Transduction coverage of european robin retina

Intravitreal ocular injection of AAV2/9 successfully transduced retinal tissue of European robins (Figures 3A–H). The transduced area ranged from 10% to 60% of the total retinal surface (Figure 3I), resulting in an overall mean transduction coverage of ∼35 ± 17% across nine individuals, which we considered consistent enough to act as a “proof of principle” for the effectiveness of the viral serotype. We observed heterogenous transduction densities of EGFP expressing neurons, with the highest numbers usually being proximal to the injection site and a gradual decrease with distance. Figures 3A, E display two exemplary 2-D reconstructions, demonstrating the retinal surface transduction coverage and its regional variability. As expected, in the non-transduced control eyes, no EGFP signal was observed, thereby validating the general functionality of the virus (data not shown).

### Types of transduced neurons in the European robin retina

We enhanced the endogenous EGFP signal with an EGFP antibody to identify the type and detailed morphology of the transduced neurons. The vast majority of EGFP reporter gene expression was found in the photoreceptor layer (Figures 3C, D, G, H). Only in very few cases, we observed EGFP expressing Müller cells, potentially resulting from occasional disruption of the inner limiting membrane (ILM) formed by Müller cell endfeet during the viral injections.

**FIGURE 4:**
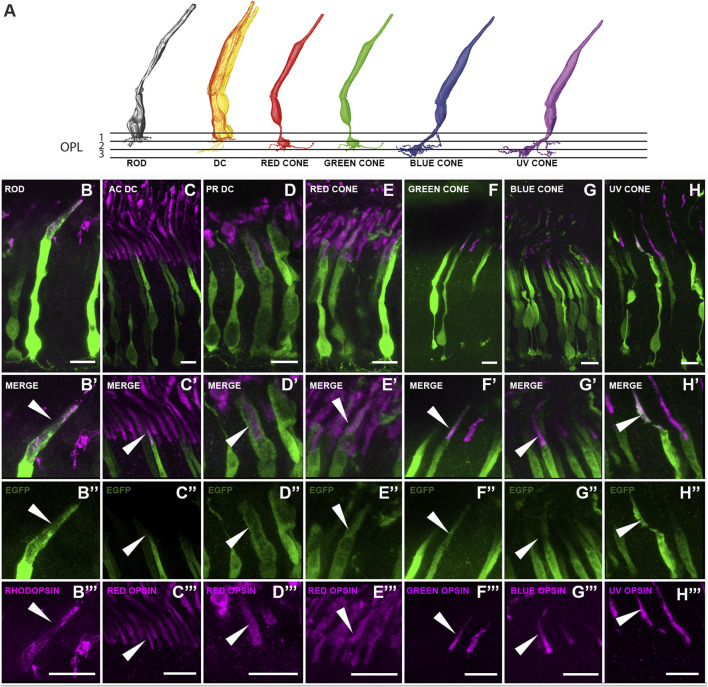
Characterization of the EGFP expressing photoreceptor types within the transduced European robin retinae. **(A)** 3-D reconstruction images based on chicken ssmSEM data (Günther et al., 2021; Günther et al., 2024) depicting the morphology of photoreceptors in the avian retina. Please note that the ssmSEM dataset could not distinguish between red-green and blue-UV cones which are presumed to have a very similar shape (Günther et al., 2021). **(B–H)** Images showing EGFP expressing photoreceptors and their outer segment localization with rhodopsin (for rods), red opsin (for accessory (AC) and principal (PR) member of double cones and red cones), green opsin (for green cones), blue opsin (for blue cones) and UV opsin (for UV cones) respectively. **(B’–H’)** Zoomed-in images showing the specific outer segment localization of the EGFP and the respective opsin. **(B’’–H’’)** EGFP expression in the outer segments. **(B’’’–H’’’)** Corresponding opsin expression. Arrowheads indicate areas of colocalization. Scale: 10 μm.

To further characterize the photoreceptor types transduced by the ocular injections, immunostainings were carried out using various opsin antibodies (Table 1; Figures 4B–H, B'–H', B''–H'', B'''–H'''). AAV2/9-EGFP targeted all photoreceptor types, i.e., rods, principal and accessory members of double cones, and the four types of single cones (red, green, blue and UV) (Figure 4). Their overall morphology largely resembled the ssmSEM-based reconstructions of chicken photoreceptors (Günther et al., 2021; Günther et al., 2024) (Figure 4A), thereby validating their morphology in the European robin: rods possessed a stout cell body and terminal with long telodendria; the accessory member of double cones was characterized by a thinner cell body with a distinct brush-like synaptic terminal located far more distal than all other terminals; the principal member of the double cones has a very broad shape and rather thick outer segment; the four single cones (red, green, blue and UV) were observed to have a thin cell body and bulb like terminal endings.

In addition to using a ubiquitous CAG promoter for seven specimens, two additional ocular injections were performed using a CAAG promoter. Both promoters resulted in a very similar high retinal transduction efficiency and variability (see blue dots in Figure 3I), thereby validating the effectiveness of the used serotype irrespective of the promoter.

## Discussion

In contrast to both the subretinal (Barker et al., 2009; Watanabe et al., 2013; Petit et al., 2017) and intravitreal route of injection (Giove et al., 2010; Reid et al., 2017), which have proven successful in transducing considerable amounts of retinal tissue in murine model systems, only the intravitreal route, due to its less invasiveness, appears feasible in avian model systems. However, so far, the intravitreal route has not yielded efficient and widespread transduction of retinal tissue in birds (Waldner et al., 2019). This could have been caused by the large relative volume of the vitreous chamber in birds and the much thicker nerve fiber layer in avian eyes, which may act as a barrier for successful transductions. The main reason, however, might have been the lack of appropriate AAV serotypes capable of successfully transducing the retina. In this study, the AAV2/9 serotype proved successful in transducing European robin retinal tissue, reaching widespread transduction coverages of ∼35 ± 17% of the total retinal surface (Figure 3I) across seven individuals. Bearing in mind that volumetric calculations indicate that European robin´s eyes are approximately 20 times larger than mouse eyes, increasing the injected volume and/or titer concentration could potentially improve the transduction efficiency even further.

In this “proof of principle” study, transduction efficiency varied between 10% and 60%. Potential reasons for this variability include 1) different amounts of viral suspension, ranging between 10 and 20 μL, 2) occasional efflux of viral suspension during injection, and 3) variable virus titers (ranging between ∼2-4 × 10^12^). Since it is well documented that a certain concentration of viruses is required to induce transduction (Giove et al., 2010; Reid et al., 2017; Waldner et al., 2019), these variations may have impacted the transduction efficiency.

Given the observed variability, future studies investigating functional aspects effects of genetic manipulations using the AAV2/9 serotype as a vehicle will require a more thorough quantitative assessment of transduction efficiency, e.g., by Western blots and/or RT-qPCR.

Avian AAVs (A3Vs) have been used for intravitreal injections in birds, but showed limited transduction efficiency (Waldner et al., 2019). In search for a suitable AAV capable to successfully transduce retinal tissue of European robins, a thorough literature survey revealed that, in contrast to AAV5 and AAV8, both AAV2 and AAV9 displayed a highly specific tropism towards various retinal cell types in mice (Lee et al., 2018). Pseudotyping, i.e., a recombinant AAV containing the structural and enzymatic component from one AAV “wrapped” in the capsid component from another, can further increase the tropism towards certain host cells. Here, we used AAV2/9-CAG-EGFP and AAV2/9-CAAG-EGFP for intravitreal delivery in the European robin retina. This serotype was chosen as it can transfect a broad range of retinal cell types, including photoreceptors and its progenitors (Allocca et al., 2007) in other vertebrate species (Watanabe et al., 2013).

We can only speculate on why AAV2/9 outperformed A3Vs in intravitreal injections in the avian retina. The vitreoretinal junction (inner limiting membrane, ILM) is a serotype specific barrier for naturally occurring AAVs in mice (Dalkara et al., 2009). It contains AAV binding sites, which create a diffusion barrier for AAV particles that arrive from the intravitreal side (Khabou et al., 2016). Thus, one potential explanation could be that the avian ILM contains more binding sites for avian AAVs (A3V) than for non-avian AAV2/9 particles, leading to the larger transduction efficiency of AAV2/9 in the avian retina. However, differences in virus titer, injection routine, or study species may also play a role.

Demonstrating the connectivity between photoreceptors and bipolar cells (Günther et al., 2021; Balaji et al., 2023; Günther et al., 2024) or horizontal cells (Günther et al., 2025) and successfully enabling photoreceptor-specific gene delivery to the European robin retina, might only be the first of many steps towards a plethora of investigations on the morphology, biochemistry and physiology of the avian retina using the AAV2/9 serotype. This is of particular importance, since birds, as mentioned before, have some of the most high-performing eyes amongst vertebrates, but so far, we have lacked the necessary tools to genetically manipulate them. Its proven functionality in European robins makes AAV 2/9 particularly well suited for studying the proposed light dependent, radical pair based magnetoreception mechanism (Hore and Mouritsen, 2016; Mouritsen, 2018; Mouritsen, 2022). This elusive sense (Zapka et al., 2009; Hore and Mouritsen, 2016; Wu et al., 2020; Wang et al., 2021; Xu et al., 2021; Görtemaker et al., 2022) is likely to be based on magnetically sensitive reactions inside the cryptochrome 4 protein (Xu et al., 2021; Chetverikova et al., 2022) located in the outer segments of double cones and long wavelength single cones (Günther et al., 2018). We are convinced that the AAV 2/9 serotype, which we identified here, will be instrumental to explore the physiology of European robin photoreceptors and the role of double cones in magnetoreception.

## Data Availability

The datasets presented in this study can be found in online repositories. The names of the repository/repositories and accession number(s) can be found below: https://www.addgene.org/29778/, Addgene plasmid #29778. The original dataset of this study will be made available from the corresponding author upon request, without undue reservation. Anti-Green opsin antibody can be obtained from Monoclonal Antibody Core Facility at Helmholtz Center Munich.
